# Using circulating tumor DNA as a novel biomarker to screen and diagnose hepatocellular carcinoma: A systematic review and meta‐analysis

**DOI:** 10.1002/cam4.2799

**Published:** 2019-12-26

**Authors:** Ziying Zhang, Peng Chen, Hui Xie, Peiguo Cao

**Affiliations:** ^1^ Department of Oncology Third Xiangya Hospital Central South University Changsha China; ^2^ Department of Urology Xiangya Hospital Central South University Changsha China; ^3^ Department of Thoracic and Cardiovascular Surgery Second Xiangya Hospital Central South University Changsha China

**Keywords:** circulating tumor DNA, diagnostic accuracy, hepatocellular carcinoma, meta‐analysis, methylation

## Abstract

**Purpose:**

A meta‐analysis was formulated to appraise the diagnostic accuracy of circulating tumor DNA (ctDNA) in hepatocellular carcinoma (HCC).

**Materials and Methods:**

We enrolled all relevant studies published until September 2019. Four primary subgroups were investigated: the subgroup of quantitative or qualitative analysis of ctDNA, the subgroup of Ras association domain family 1 isoform A （*RASSF1A*） methylation in ctDNA and the subgroup of the combined alpha‐fetoprotein (AFP) and ctDNA assay. We analyzed the pooled sensitivity (SEN), specificity (SPE), positive likelihood ratio (PLR), negative likelihood ratio (NLR), diagnostic odds ratio (DOR), and summary receiver operating characteristic (SROC) as well as the area under the curve (AUC).

**Results:**

A total of 33 qualified articles with 4113 subjects were incorporated into our meta‐analysis. The combined SEN, SPE, and DOR in quantitative studies were 0.722 (95% confidence interval (95% CI): 0.686‐0.756), 0.823 (95% CI: 0.789‐0.854), 18.532 (95% CI: 8.245‐41.657), respectively, yielding an AUC of 0.880. For qualitative studies, the corresponding value was 0.568 (95% CI: 0.548‐0.587), 0.882 (95% CI: 0.867‐0.897), 10.457 (95% CI: 7.270‐15.040) and 0.787, respectively. Detection of *RASSF1A* methylation yielded an AUC of 0.841, with a SEN of 0.644 (95% CI: 0.608‐0.678) and a SPE of 0.875 (95% CI: 0.847‐0.900). AFP combined with ctDNA assay achieved an AUC of 0.944, with a SEN of 0.760 (95% CI: 0.728‐00.790) and a SPE of 0.920 (95% CI: 0.893‐00.942).

**Conclusion:**

Circulating tumor DNA displays a promising diagnostic potential in HCC. However, it is not independently sufficient and can serve as an assistant tool combined with AFP for HCC screening and detection.

## Graphical abstract

We performed the first integrated meta‐analysis on the overall diagnostic accuracy of circulating tumor DNA assays in HCC. Quantitative and qualitative analysis of ctDNA can be used as a complementary strategy integrated with AFP assay for the early detection and diagnosis of HCC.

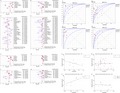

## 1 INTRODUCTION

Liver cancer, with over 841 000 patients globally, has currently become the second most frequent reason for tumor‐related deaths.^1^, ^2^ Hepatocellular carcinoma (HCC), as the most common pathologic subtype of primary liver tumors, occupies approximately 90% of all patients.^2^, ^3^ The prognosis of untreated HCC patients is undesirable with a median survival of 2‐14 months.^2^, ^4^, ^5^ Compelling observational data have demonstrated that earlier HCC detection and therapeutic interventions are conducive to boosting the overall survival of patients.^6^

Currently, surgical intervention, such as partial hepatic resection and hepatic transplantation remain the primary therapeutic strategies for HCC patients. Indeed, if patients with early HCC that is currently hard to recognize and delineate could be accurately diagnosed, the 5‐year survival rate for HCC patients who have received surgery would reach up to 90%.^7^ Unfortunately, a large proportion of HCC individuals are usually diagnosed at an advanced stage on account of the non‐specific clinical symptoms and the limitations in detection methods, thus triggering that fewer than 30% of the patients are qualified for surgical treatment.^8^, ^9^ Early screening for HCC has been conducted in several cohorts following the Asian‐Pacific Association for the Study of the Liver guidelines, which advocates that HCC surveillance should be implemented for clinical subjects with liver cirrhosis and those with positive surface antigen of hepatitis B virus (HBsAg) by utilizing liver ultrasonography (US) and serum alpha‐fetoprotein (AFP) test every 6 months.^10^

Nevertheless, the diagnostic efficiency of AFP assay for HCC is not satisfactory, with a sensitivity (SEN) of 25%‐65% and a specificity (SPE) lower than 82%, respectively.^11^ When liver US is applied for the detection of HCC nodules smaller than one cm, its SEN is approximately 60%.^12^ Additionally, the fluctuation in AFP levels is also associated with inflammation and liver disease type.^13^ For example, AFP levels may be enhanced in non‐HCC conditions, including chronic liver diseases (such as liver cirrhosis and hepatic inflammation), other tumors (such as intrahepatic cholangiocarcinoma and metastatic colon cancer) as well as pregnancy.^14^, ^15^, ^16^ Therefore, the detection of HCC with these methods remains suboptimal, it is imperative to develop additional biomarkers for early detection and diagnosis of HCC in a minimally invasive, convenient and accurate manner.

Accumulating evidence has indicated that the cumulation of genetic and epigenetic changes in liver tissue results in the tumorigenesis and development of HCC, which is intimately associated with the surrounding microenvironment.^17^ Recent progresses in the field have highlighted that the minimally invasive detection of circulating tumor DNA (ctDNA) confers a promising opportunity for the early screening and diagnosis of HCC. This assay, in conjunction with circulating tumor cells and circulating cell‐free DNA, is termed “liquid biopsy”.^18^ Circulating tumor DNA is generally derived from apoptotic or necrotic tumor cells and further released into the circulation,^19^ which carries cancer‐specific modifications in gene or epigenetics, including single nucleotide mutation,^20^ copy number aberration (CNA),^21^ and DNA methylation^19^ or 5‐hydroxymethylcytosines.^16^ Quantitative alteration and qualitative alteration of ctDNA are primarily detected in HCC patients. The former is associated with measuring the quantity of ctDNA that is generally increased in HCC patients,^22^ and the latter refers to monitoring tumor‐specific genetic aberrations. Specifically, with the booming development of next generation sequencing, a growing number of studies have been concentrating on the “methylation pattern” of ctDNA in HCC patients and demonstrated that tumor‐specific alterations in methylation may represent a novel discriminatory tool for the screening, detection, and diagnosis of HCC. Thus, by deciphering the information of deoxyribonucleic acid derived from HCC patients’ circulation, clinicians can utilize this “liquid biopsy” technology to confer precise diagnosis and appropriate therapy for HCC patients.

Although a considerable number of studies have revealed the diagnostic efficiency of ctDNA for HCC, the results are very diverse partially ascribed to the discrepancies in study design and assay methods for ctDNA among studies. Thus, prior to its clinical utilization, a comprehensive analysis and evaluation of the diagnostic value of ctDNA in HCC is imminently required. Herein, we implemented a meta‐analysis to objectively assess the diagnostic performance of ctDNA assays for HCC, which potentially confers guideline for technology improvement and clinical applications.

## 2 MATERIALS AND METHODS

### 2.1 Search strategy

All potentially relevant articles that were published up to September 2019 were retrieved and the following electronic databases were independently queried by two authors: PubMed, Web of Science, Embase, Cochrane Library, and China National Knowledge Infrastructure. The query terms were as follows: “circulating tumor DNA” OR “circulating DNA” OR “ctDNA” OR “plasma DNA” OR “serum DNA” OR “blood DNA” AND “liver cancer” OR “hepatocellular carcinoma” OR “liver neoplasms” OR “hepatic carcinoma” OR “liver tumor” AND “diagnosis” OR “sensitivity” OR “specificity” OR “accuracy”. The language of all articles was limited to English. We also manually screened the reference from the included articles and relevant reviews for enlarged retrieval.

### 2.2 Inclusion and exclusion criteria

The publications that conformed to the following criteria were incorporated: (a) ctDNA indicators were used for the first diagnosis rather than the recurrent diagnosis of HCC; (b) the numerical value of SEN and SPE could be collected either directly from the papers or could be calculated in each study; and (c) specimens were extracted from peripheral blood. The exclusion criteria were as follows: (a) review, case report, letter or conference abstract; (b) the sample size of studies was less than 10; and (c) duplicate or overlapping publications that included the same population and gene. Two authors independently evaluated the eligibility of studies. Discrepancies were resolved via consensus.

### 2.3 Data extraction

Two authors independently conducted data extraction from the included studies and further summarized the ultimate results. The information extracted from the incorporated publications was as follows: the first author's name, publication year, region/country, study design, participant characteristics (including sample size, control type), detection details (including source of specimens, sampling time, experimental methods, reference gene, cutoff values), diagnostic performance (including SEN and SPE, true positive (TP), true negative (TN), false positive (FP), and false negative (FN), positive likelihood ratio (PLR) and negative likelihood ratio (NLR), and diagnostic concordance).

### 2.4 Quality assessment

Based on the revised Quality Assessment of Diagnostic Accuracy Studies‐2 (QUADAS‐2), the risk of bias and concerns about applicability of all included publications were evaluated as “low risk”, “high risk” and “unclear risk” through four key domains including patient selection, index test, reference standard, and flow and timing.^23^ All the studies were independently assessed and rated by two authors. Divergence was discussed until an agreement was reached. If an article was evaluated to be of poor quality by two authors, it would be excluded.

### 2.5 Statistical analysis

We utilized RevMan Manager 5.3 and Meta‐Disc 1.4 software to conduct this diagnostic meta‐analysis. The pooled SEN and SPE, PLR, NLR, diagnostic odds ratio (DOR) and corresponding 95% confidence interval (95% CI) were calculated as evaluation indicators.^24^ Simultaneously, the summary receiver operating characteristic (SROC) curve and its corresponding area under the curve (AUC) value were formulated to evaluate the overall test accuracy.^25^, ^26^, ^27^, ^28^ The closer the AUC value was to 1, the higher was the diagnostic efficiency.^29^, ^30^ The AUC range of 0.5‐0.7, 0.7‐0.9, 0.9‐1.0 corresponded to low, moderate or high accuracy, respectively.^29^ The Spearman correlation coefficient and its corresponding P value were used to identify the presence of the threshold effect. Generally, threshold effect was considered to exist when the P value was lower than 0.05. If heterogeneity resulted from nonthreshold effect, we utilized the chi‐square and I^2^ test to evaluate the heterogeneity among the studies. *I* ^2^ > 50% or *P* < .05 suggested a significant heterogeneity.^24^, ^31^ Thus, the random effect model was applied for statistical analysis. Further subgroup analysis and meta‐regression analysis were also conducted to probe the source of heterogeneity.^32^ The Deek's funnel plot was formulated to examine the existence of potential publication bias.^33^ A result with *P* value < .05 was considered to be statistically significant.

## 3 RESULTS

### 3.1 Study characteristics

**Figure 1:**
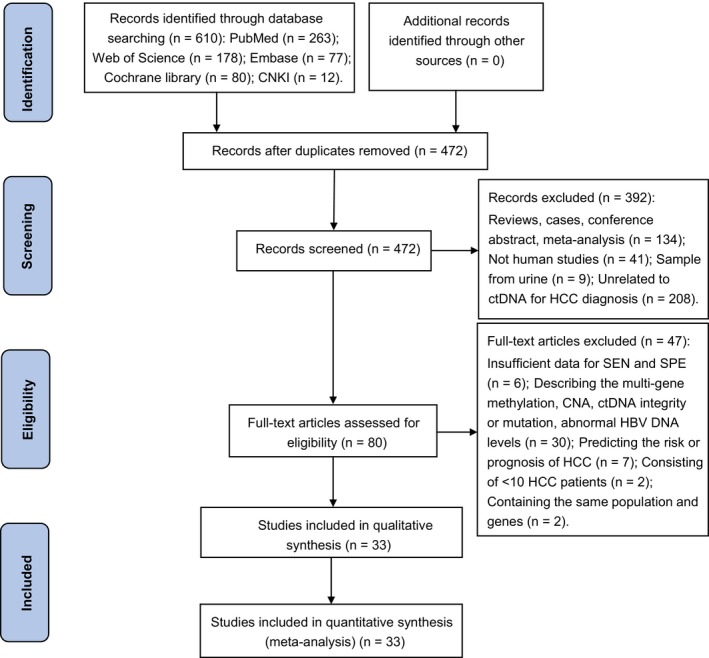
A PRISMA flow diagram of the literature search. CAN, copy number aberration; CNKI: China National Knowledge Infrastructure; ctDNA: circulating tumor DNA; HBV: hepatitis B virus; HCC, hepatocellular carcinoma; PRISMA, Preferred Reporting Items for Systematic Reviews and Meta‐Analyses; SEN, sensitivity; SPE, specificity

Figure 1 shows a Preferred Reporting Items for Systematic Reviews and Meta‐Analyses (PRISMA) flow diagram adapted from Moher et al^34^ depicting the retrieval strategy of databases to incorporate qualified publications. Initially, a total of 610 publications were queried through our search strategy. Eventually, 33 eligible articles^22^, ^35^, ^36^, ^37^, ^38^, ^39^, ^40^, ^41^, ^42^, ^43^, ^44^, ^45^, ^46^, ^47^, ^48^, ^49^, ^50^, ^51^, ^52^, ^53^, ^54^, ^55^, ^56^, ^57^, ^58^, ^59^, ^60^, ^61^, ^62^, ^63^, ^64^, ^65^, ^66^ published from 2000 to 2019 were incorporated into this diagnostic meta‐analysis following the exclusion of duplicate studies, the examination of title and abstract as well as the comprehension of full text. Specifically, all the included studies consisted of quantitative analysis to measure ctDNA concentration (n = 5)^22^, ^56^, ^57^, ^58^, ^59^ and qualitative analysis to unravel tumor‐specific single‐gene methylation in ctDNA (n = 25),^35^, ^36^, ^37^, ^38^, ^39^, ^40^, ^41^, ^42^, ^43^, ^44^, ^45^, ^46^, ^47^, ^48^, ^49^, ^50^, ^51^, ^52^, ^53^, ^54^, ^55^, ^63^, ^64^, ^65^, ^66^ as well as both quantitative and qualitative analysis (n = 3).^60^, ^61^, ^62^ Among these 33 publications, 11 articles described the diagnostic role of circulating Ras association domain family 1 isoform A (*RASSF1A*) methylation in HCC^37^, ^39^, ^40^, ^48^, ^49^, ^50^, ^52^, ^60^, ^61^, ^62^, ^65^ and eight articles evaluated the diagnostic performance of ctDNA combined with AFP assay in HCC.^38^, ^40^, ^41^, ^42^, ^43^, ^44^, ^54^, ^56^ Nine articles assessed the diagnostic accuracy of AFP assay for HCC 37, 40, 41, 42, 43, 44, 62, 63, 64

Our study enrolled a total population of 2268 HCC patients and 1845 control individuals (1318 patients with benign liver disorders and 527 healthy volunteers). The primary characteristics of all participants are summarized in Table 1. An overwhelming majority of participants were Asian (n = 3808), with the residual 160 participants from America and 145 individuals from Egypt. Twelve researches belonged to retrospective (n = 8) or prospective trial (n = 4), respectively, and the remaining publications (n = 21) did not definitely describe the study design. Among 19 studies with known time point of sampling, most of the samples were collected before treatment (n = 14) and ctDNA was obtained from plasma (n = 13), serum (n = 18), both plasma and serum (n = 2). The assay methods to measure the concentrations of ctDNA were real‐time quantitative polymerase chain reaction (RT‐qPCR) (n = 5), ultraviolet transilluminator (n = 1), enzyme‐linked immunosorbent assay (ELISA) (n = 1) and droplet digital PCR DNA (DdPCR) (n = 1). For qualitative analysis of ctDNA, methylation‐specific polymerase chain reaction (MSP) was the most common method used (n = 25).

**Table 1:** Summary of the most relevant features of the enrolled publications

First author, year	Country/Region	Study type	Control type	No. of HCC/BD/HC	Timing sample	Sample source	Detection methods	Assay indicators	Cutoff value	SEN (%)	SPE (%)	PLR	NLR	Diagnostic concordance	TP	FP	FN	TN
Kisiel, 2019	USA	Retrospective	LC	21/30/‐	During surgery	Plasma	MSP	Methylation (*EMX1*)	NA	76.0	100.0	Infinity	0.240	0.902	16	0	5	30
Wei, 2018	China	NA	BD/HC	119/157/50	Pre surgery	Plasma	MSP	Methylation (*SOCS3*)	NA	28.6	95.2	5.958	0.750	0.709	34	10	85	197
Dong, 2017	China	NA	CHB and LC/HC	98/165/80	NA	Serum	MSP	Methylation (*RASSF1A*)	NA	52.0	93.1	7.536	0.516	0.813	51	17	47	228
Mansour, 2017	Egypt	NA	HCV infection	45/40/‐	NA	Serum	MSP	Methylation (*RASSF1A*)	8 copies/µL	86.7	72.5	3.153	0.183	0.800	39	11	6	29
Hu, 2017	China	NA	LC, CHB	80/80/‐	Pre treatment	Serum	MSP	Methylation (*UBE2Q1*)	NA	66.3 [53.8] **§**	57.5 [87.5]	1.560 [4.30]	0.586 [0.53]	0.619 [0.706]	53 [43]	34 [10]	27 [37]	46 [70]
Huang, 2015	China	Retrospective	LC	31/10/‐	NA	Plasma	MSP	Methylation (*RASSF1A*)	NA	51.6	80.0	2.580	0.605	0.585	16	2	15	8
								Methylation (*GSTP1*)	NA	38.7	60.0	0.968	1.022	0.439	12	4	19	6
								Methylation (*P16*)	NA	41.9	70.0	1.397	0.830	0.488	13	3	18	7
Dong, 2015	China	NA	CHB	190/120/‐	Pre treatment	Serum	MSP	Methylation (*RASSF1A*)	NA	64.2 [80.9]	89.8 [93.4]	6.294 [12.3]	0.399 [0.20]	0.742 [0.855]	122 [153]	12 [8]	68 [37]	108 [112]
Li, 2014	China	NA	CHB	136/46/‐	Pre treatment	Serum	MSP	Methylation (*IGFBP7*)	NA	65.4	82.6	3.759	0.419	0.698	89	8	47	38
Huang, 2014	USA	NA	BD	66/43/‐	NA	Serum	Pyrosequencing	Methylation (*INK4A*)	5%¶	65.3 [80.3]	87.2 [100]	5.102 [Infinity]	0.398 [0.20]	0.734 [0.881]	43 [53]	6 [0]	23 [13]	37 [43]
Han, 2014	China	Retrospective	CHB	160/88/‐	NA	Serum	MSP	Methylation (*TGR5*)	NA	48.1 [65.0]	86.4 [85.2]	3.537 [4.39]	0.601 [0.41]	0.617 [0.722]	77 [104]	12 [13]	83 [56]	76 [75]
Yang, 2014	China	NA	LC and CHB	123/57/‐	NA	Serum	MSP	Methylation (*IDO1*)	NA	42.3 [83.0]	89.5 [87.7]	4.029 [6.75]	0.645 [0.19]	0.572 [0.844]	52 [102]	6 [7]	71 [21]	51 [50]
Ji, 2014	China	Retrospective	CHB	100/37/‐	Pre surgery	Serum	MSP	Methylation (*MT1M*)	NA	50	94.6	9.259	0.529	0.533	50	14	50	23
								Methylation (*MT1G*)	NA	69	83.8	4.259	0.370	0.730	69	6	31	31
Kuo, 2014	Taiwan	NA	HC	39/‐/34	During surgery	Plasma	MSP	Methylation (*HOXA9*)	>0.88**†**	73.3 [94.6]	97.1 [97.1]	25.28 [32.6]	0.275 [0.06]	0.849 [0.959]	29 [37]	1 [1]	10 [2]	33 [33]
Zhang, 2013	China	NA	HC	31/‐/27	Pre treatment	Serum	Chip/Pyrosequencing	Methylation (*DBX2*)	NA	88.9	87.1	6.891	0.127	0.897	28	3	3	24
								Methylation (*THY1*)	NA	85.2	80.7	4.415	0.183	0.828	26	5	5	22
Sun, 2013	China	NA	CHB	43/24/‐	NA	Serum	MSP	Methylation (*TFPI2*)	NA	46.5	83.3	2.784	0.642	0.597	20	4	23	20
Mohamed, 2012	Egypt	Case‐control	HC	40/‐/20	NA	Serum	MSP	Methylation (*RASSF1A*)	304 nmol/L	75.0	80.0	3.750	0.313	0.767	30	4	10	16
Lizuka, 2011	Japan	Prospective	HCV infection	108/56/‐	NA	Serum	MSP	Methylation (*SPINT2*)	0.2 pg/mL	35.2	98.2	19.56	0.660	0.567	38	1	70	55
								Methylation (*RASSF1A*)	0.2 pg/mL	83.3	58.9	2.027	0.284	0.750	90	23	18	33
Huang, 2011	China	NA	BD	72/‐/41	Pre treatment	Plasma	MSP	Methylation (*APC*)	NA	68.1	97.6	28.38	0.327	0.788	49	1	23	40
								Methylation (*GSTP1*)	NA	55.6	90.2	5.673	0.492	0.681	40	4	32	37
								Methylation (*RASSF1A*)	NA	72.2	95.1	14.74	0.292	0.805	52	2	20	39
Sun, 2010	Hong Kong	Retrospective	LC/HC	35/16/12	NA	Plasma	RT‐qPCR	Methylation (*LMNB1*)	NA	80.0	82.0	4.444	0.244	0.810	28	5	7	23
Hu, 2010	China	Retrospective	HC	35/‐/10	Pre surgery	Serum	MSP	Methylation (*RASSF1A*)	NA	40.0	100.0	Infinity	0.600	0.533	14	0	21	10
Chang, 2008	China	NA	LC	26/16/‐	NA	Plasma	MSP	Methylation (*RASSF1A*)	NA	26.9	81.3	1.439	0.899	0.476	7	3	19	13
Zhang, 2007	Taiwan	Prospective	HC	50/‐/50	Closest to diagnosis	Serum	MSP	Methylation (*P16*)	NA	44.0	96.0	11.00	0.583	0.700	22	2	28	48
								Methylation (*P15*)	NA	22.0	100.0	Infinity	0.780	0.610	11	0	39	50
								Methylation (*RASSF1A*)	NA	70.0	94.0	11.67	0.319	0.820	35	3	15	47
Wang, 2006	China	NA	LC	32/8/‐	NA	Serum	MSP	Methylation (*GSTP1*)	NA	50.0	62.5	1.333	0.800	0.525	16	3	16	5
Yeo, 2005	Hong Kong	NA	HC	40/‐/10	Pre surgery	Plasma	MSP	Methylation (*RASSF1A*)	NA	42.5	100.0	Infinity	0.575	0.540	17	0	23	10
Lin, 2005	China	NA	BD/HC	64/15/20	Pre‐ and post‐surgery	Serum	MSP	Methylation (*p16*)	NA	76.6	100.0	Infinity	0.234	0.848	49	0	15	35
Chu, 2004	Korea	NA	LC	46/23/‐	NA	Serum	MSP	Methylation (*p16INK4a*)	NA	47.8	82.6	2.747	0.632	0.594	22	4	24	19
Wong, 2003	Hong Kong	Prospective	BD/HC	45/30/20	Pre‐, intra‐ and post‐surgery	Serum/plasma	MSP	Methylation (*p16INK4a*)	NA	31.1 [62.0]	100.0 [100]	Infinity [Infinity]	0.689 [0.38]	0.674 0.821	14 [28]	0 [0]	31 [17]	50 [50]
Wong, 2000	Hong Kong	Prospective	BD/HC	25/35/20	Pre surgery	Serum/plasma	MSP	Methylation (*p16*)	NA	60.0	100.0	Infinity	0.400	0.594	15	0	10	55
Gai, 2018	Hong Kong	NA	HBV carriers and LC/HC	40/29/30	NA	Plasma	DdPCR	ctDNA	370 copies/mL	93.0	60.0	2.325	0.117	0.674	37	24	3	35
Huang, 2012	China	NA	HC	72/‐/41	Pre treatment	Plasma	RT‐qPCR	ctDNA	18.2 ng/mL	90.2 [95.1]	90.3 [94.4]	9.299 [17.0]	0.109 [0.05]	0.903 [0.973]	65 [71]	4 [2]	7 [1]	37 [39]
Yang, 2011	China	case control	HBV infection/HC	60/21/29	Pre treatment	Plasma	RT‐qPCR	*hTERT*	1.87 × 10^4^ copies/uL	64.0	90.0	6.400	0.400	0.903	38	5	22	45
Dong, 2008	China	NA	LC, CH and AH/HC	117/152/40	Pre surgery	Plasma	ELISA	*TGF‐β1*	1.2 μg/L	89.7	91.1	10.08	0.113	0.755	105	17	12	175
Ren, 2006	China	NA	LC/HC	79/20/20	Pre surgery	Plasma	Ultraviolet transilluminator	ctDNA	36.6 ng/mL	51.9	77.5	2.307	0.621	0.906	41	9	38	31

Abbreviations: AH, acute hepatitis; *CDO1*, Cysteine dioxygenase 1; CH, chronic hepatitis; CHB, Chronic hepatitis B; CLD, chronic liver diseases; DdPCR, droplet digital PCR; ELISA, enzyme‐linked immunosorbent assay; *EMX1*, empty spiracles homeobox 1; FN, false negative; FP, false positive; GPC‐3, glypican‐3; HBV, hepatitis B virus; HCV, hepatitis C virus; *IGFBP7*, insulin‐like growth factor‐binding protein 7; LC, liver cirrhosis; *LMNB1*, Lamin B1; MSP, methylation‐specific polymerase chain reaction; NA, not applicable; NLR, negative likelihood ratio; No. of HCC/BD/HC, number of hepatocellular carcinoma/benign live diseases/ healthy controls; *RASSF1A*, Ras association domain family 1 isoform A; PLR, positive likelihood ratio; RT‐qPCR, real‐time quantitative polymerase chain reaction; SEN, sensitivity; SPE, specificity; *TGR5*:, G‐protein‐coupled bile acid receptor Gpbar1; TN, true negative; TP, true positive.

^§^ The SEN and SPE, PLR and NLR, diagnostic concordance, TP, TN, FP and FN of the ctDNA combined with AFP for HCC detection are presented in [].

^¶^ A limit of detection (LOD) of 5%.

^†^ Methylation index.

### 3.2 Quality assessment

**Figure 2:**
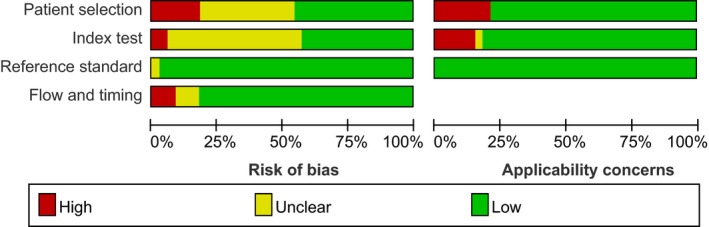
Quality assessment of the included studies by the revised QUADAS‐2. QUADAS‐2, Quality Assessment of Diagnostic Accuracy Studies‐2

The quality assessment outcome of the eligible 33 publications is revealed in Figure 2 and Figure S1. A majority of the literature exhibited a moderate‐high quality, indicating that the overall quality of the included studies was commonly robust. Nevertheless, 13 studies might generate an unknown risk of bias in index test because of insufficient information about predefined threshold. Additionally, 12 studies did not mention whether the patient selection was consecutive or random in a particular time period, potentially generating an unknown risk of performance bias. All enrolled patients obtained definite pathological diagnoses.

### 3.3 Diagnostic accuracy

#### 3.3.1 Diagnostic value of quantitative and qualitative analysis of ctDNA for HCC

**Figure 3:**
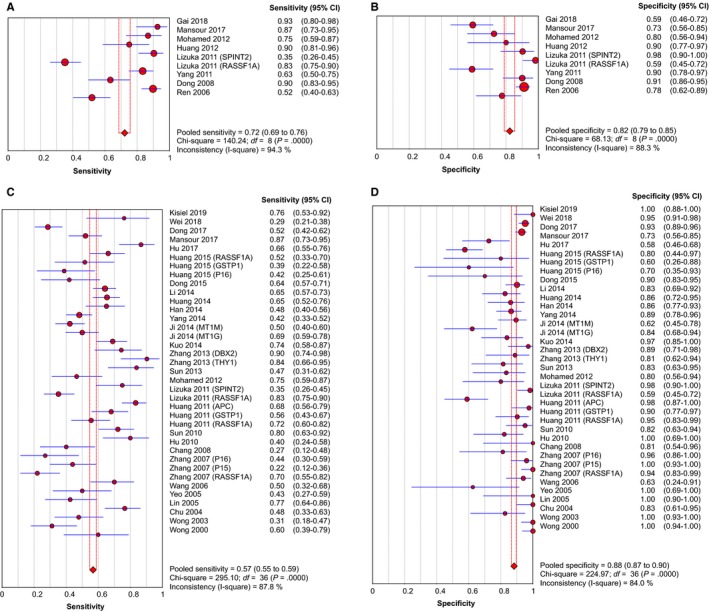
Forest plots of SEN and SPE for diagnostic value of ctDNA assay for HCC in (A) the quantitative detection subgroup and (B) the qualitative detection subgroup with the diagnostic indicator of cancer‐specific single‐gene methylation alterations in ctDNA. ctDNA, circulating tumor DNA; HCC, hepatocellular carcinoma; SEN, sensitivity; SPE, specificity

The quantitative detection of ctDNA discriminated HCC patients from control individuals with a SEN of 0.722 (95% CI: 0.686‐0.756) and a SPE of 0.823 (95% CI: 0.789‐0.854) (Figure 3A,B). The numerical value of PLR, NLR and DOR was 4.208 (95% CI: 2.526‐7.009), 0.264 (95% CI: 0.145‐0.483), 18.532 (95% CI: 8.245‐41.657), respectively. This also corresponded to the SROC curve with an AUC of 0.880 (Figure 5A), indicating a higher level of moderate overall accuracy to differentiate HCC patients from control subjects. Among the included quantitative studies, there was significant heterogeneity (SEN: *I* ^2^ = 94.3%, *P* = .000; SPE: *I* ^2^ = 88.3%, *P* = .000; DOR: *I* ^2^ = 80.7%, *P* = .000) and the Spearman correlation coefficient was 0.283 (*P* = .460), indicating that heterogeneity among studies was derived from nonthreshold effects.

Similarly, in the qualitative analysis group associated with tumor‐specific single‐gene methylation, a SEN of 0.568 (95% CI: 0.548‐0.587), a SPE of 0.882 (95% CI: 0.867‐0.897) and a DOR of 10.457 (95% CI: 7.270‐15.040) were revealed (Figure 3C,D). The combined PLR and NLR were 4.378 (95% CI: 3.251‐5.897), 0.489 (95% CI: 0.431‐0.555), respectively. It further exhibited an AUC of 0.787 in SROC curve, highlighting acceptably moderate levels of diagnostic accuracy of ctDNA for HCC (Figure 5B). Significant heterogeneity was also revealed in the diagnostic analysis of qualitative studies (SEN: *I* ^2^ = 87.8%, *P* = .000; SPE: *I* ^2^ = 84.0%, *P* = .000; DOR: *I* ^2^ = 68.8%, *P* = .000). There was no significant threshold effect because of the Spearman correlation coefficient of 0.168 and the *P* value of .320.

#### 3.3.2 Diagnostic value of ctDNA combined with AFP assay for HCC

**Figure 4:**
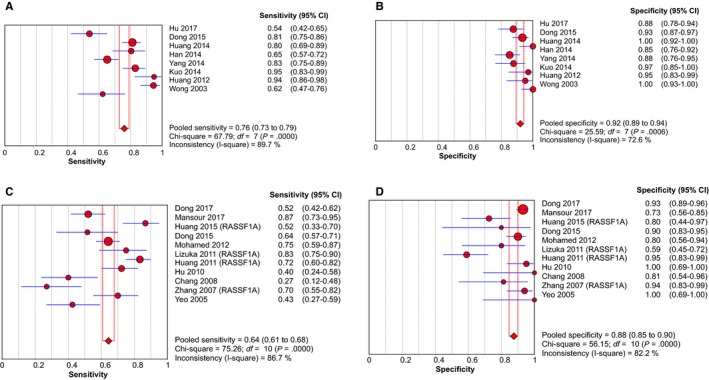
Forest plots of SEN and SPE for diagnostic value of ctDNA assay for HCC in (A) the subgroup of ctDNA combined with AFP assay and (B) the RASSF1A methylation detection subgroup. AFP, alpha‐fetoprotein; ctDNA: circulating tumor DNA; HCC: hepatocellular carcinoma; SEN: sensitivity; SPE: specificity

**Figure 5:**
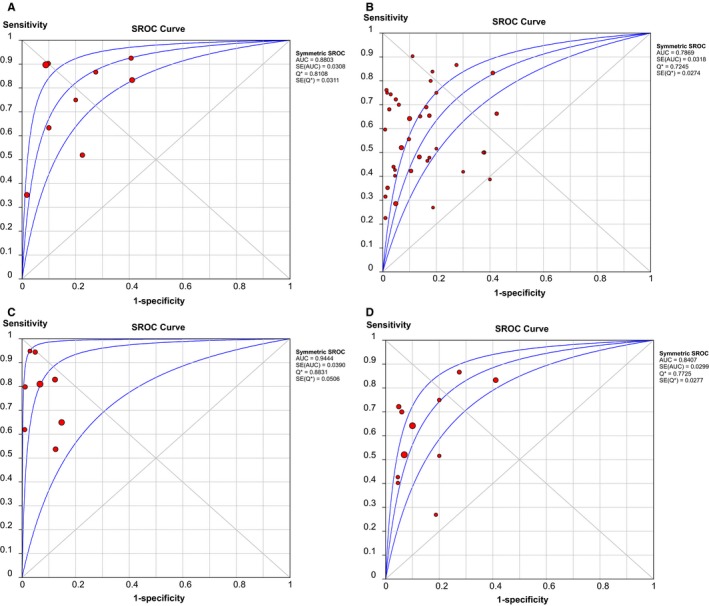
SROC curves of diagnostic value for (A) the quantitative detection subgroup; (B) the qualitative detection subgroup; (C) the subgroup of ctDNA combined with AFP assay; and (D) the RASSF1A methylation detection subgroup. AFP, alpha‐fetoprotein; AUC, area under the curve; ctDNA: circulating tumor DNA; SROC, summary receiver operating characteristic.

Initially, we evaluated the diagnostic efficiency of AFP assay in HCC. The AFP test yielded an AUC of 0.638, with a SEN of 0.478 (95% CI: 0.447‐0.509) and a SPE of 0.840 (95% CI: 0.809‐0.867) (Figure S2). The value of PLR, NLR and DOR were 3.368 (95% CI: 1.913‐5.929), 0.611 (95% CI: 0.506‐0.738), and 6.284 (95% CI: 3.109‐12.700), respectively. Furthermore, the combination of ctDNA and AFP assay yielded an AUC of 0.944 (Figure 5C), with a SEN of 0.760 (95% CI: 0.728‐0.790) and a SPE of 0.920 (95% CI: 0.893‐0.942) (Figure 4A,B). This corresponded to a PLR of 9.469 (95% CI: 5.178‐17.313), an NLR of 0.234 (95% CI: 0.154‐0.357) and a DOR of 54.864 (95% CI: 19.980‐150.66), highlighting that compared with the ctDNA assay or AFP test alone, the detection of ctDNA integrated with AFP could distinguish HCC patients from control individuals with a remarkably increased high level of accuracy (Table 2).

**Table 2:** Summary of diagnostic accuracy of ctDNA assay for HCC in multiple subgroups

Group	SEN (95% CI)	SPE (95% CI)	PLR (95% CI)	NLR (95% CI)	DOR (95% CI)	AUC (95% CI)
AFP assay	0.478 (0.447‐0.509)	0.840 (0.809‐0.867)	3.368 (1.913‐5.929)	0.611 (0.506‐0.738)	6.284 (3.109‐12.700)	0.638
Quantitative analysis of ctDNA	0.722 (0.686‐0.756)	0.823 (0.789‐0.854)	4.208 (2.526‐7.009)	0.264 (0.145‐0.483)	18.532 (8.245‐41.657)	0.880
Qualitative analysis of ctDNA	0.568 (0.548‐0.587)	0.882 (0.867‐0.897)	4.378 (3.251‐5.897)	0.489 (0.431‐0.555)	10.457 (7.270‐15.040)	0.787
AFP combined with ctDNA	0.760 (0.728‐0.790)	0.920 (0.893‐0.942)	9.469 (5.178‐17.313)	0.234 (0.154‐0.357)	54.864 (19.980‐150.66)	0.944

Abbreviations: 95% CI, 95% confidence interval; AFP, alpha‐fetoprotein; AUC: the area under the curve; DOR, diagnostic odds ratio; ctDNA, circulating tumor DNA; HCC, hepatocellular carcinoma; NLR, negative likelihood ratio; PLR, positive likelihood ratio; SEN, sensitivity; SPE, specificity.

#### 3.3.3 Diagnostic value of circulating *RASSF1A* methylation for HCC

In the qualitative analysis of ctDNA, circulating *RASSF1A* promoter methylation is the most frequently detected epigenetic change in HCC. Thus, we also estimated the diagnostic efficacy of *RASSF1A* methylation in discriminating HCC patients from controls. In the 11 studies describing circulating *RASSF1A* methylation, the pooled SEN and SPE was 0.644 (95% CI: 0.608‐0.678) and 0.875 (95% CI: 0.847‐0.900), respectively (Figure 4C,D). The pooled PLR and NLR was 4.525 (95% CI: 2.757‐7.426) and 0.439 (95% CI: 0.345‐0.557), respectively, and the DOR was 12.550 (95% CI: 7.826‐20.126). The AUC for *RASSF1A* was 0.841 (Figure 5D), indicating that ctDNA *RASSF1A* methylation can be considered as a potential HCC diagnostic biomarker with a higher level of moderate overall accuracy.

### 3.4 Subgroup analysis and meta‐regression analysis

Subgroup analysis was performed based on different covariates: region (Asia vs non‐Asia), sample size (≥100 vs <100), control type (benign disease vs healthy controls), sample source (plasma vs serum), assay methods (RT‐qPCR vs other methods in the quantitative study; MSP vs other methods in the qualitative studies) and methylation gene location (*RASSF1A* vs other gene targets in the qualitative studies) (Table 3). For the quantitative analysis of ctDNA, subgroup analyses based on sample source revealed that compared with sample collected from serum, sampling from plasma achieved an increased diagnostic accuracy in discriminating HCC from control subjects, with SEN of 0.777 (95% CI: 0.731‐0.819) vs 0.654 (95% CI: 0.598‐0.708), and SPE of 0.846 (95% CI: 0.805‐0.880) vs 0.773 (95% CI: 0.703‐0.834) as well as an AUC of 0.902 vs 0.843, respectively. Another subgroup analysis associated with control type showed that the SEN, SPE and AUC for quantitative ctDNA assay to distinguish HCC patients from healthy subjects was 0.775 (95% CI: 0.731‐0.814), 0.843 (95% CI: 0.804‐0.877) and 0.895, respectively. While the corresponding indicators to discriminate HCC from benign liver diseases were much lower at 0.697 (95% CI: 0.657‐0.735), 0.817 (95% CI: 0.780‐0.851) and 0.868, respectively. Similarly, in term of subgroup analysis related to control type in the included qualitative analysis of ctDNA, studies using healthy controls were characterized with more satisfactory diagnostic efficiency compared with those utilizing subjects with benign liver disorders, displaying SEN of 0.604 (95% CI: 0.563‐0.644) vs 0.556 (95% CI: 0.533‐0.579) and SPE of 0.938 (95% CI: 0.916‐0.955) vs 0.852 (95% CI: 0.831‐0.872), respectively. These results highlighted a more robust capability of the ctDNA assay to differentiate HCC patients from healthy individuals than from benign patients. We also performed the meta‐regression analysis to further explore the source of heterogeneity. As has been revealed in Table 4, the parameter of “control type” potentially was the primary source of heterogeneity in the qualitative analysis group (*P* = .022). None of parameters might generate significant heterogeneity in the quantitative analysis group (both *P* > .05).

**Table 3:** Subgroup analysis of diagnostic performance of ctDNA assay for HCC

Analysis	Group	Subgroup	SEN (95% CI)	SPE (95% CI)	DOR (95% CI)	AUC
Quantitative analysis	Control type	HC	0.775 (0.731‐0.814)	0.843 (0.804‐0.877)	21.320 (6.848‐66.377)	0.895
		BD	0.697 (0.657‐0.735)	0.817 (0.780‐0.851)	16.015 (6.334‐40.496）	0.868
	Sample size	≥100	0.693 (0.652‐0.732)	0.864 (0.829‐0.895)	20.501 (6.323‐66.466)	0.887
		˂100	0.848 (0.773‐0.906)	0.672 (0.580‐0.756)	15.676 (7.740‐31.750)	0.855
	Sample source	Plasma	0.777 (0.731‐0.819)	0.846 (0.805‐0.880)	23.762 (6.321‐89.324)	0.902
		Serum	0.654 (0.598‐0.708)	0.773 (0.703‐0.834)	10.632 (6.199‐18.236)	0.843
	Assay method	RT‐qPCR	0.693 (0.647‐0.736)	0.817 (0.765‐0.862)	17.568 (8.502‐36.304）	0.873
		Other methods	0.775 (0.717‐0.827)	0.828 (0.780‐0.870)	18.307 (2.271‐147.58)	0.875
Qualitative analysis	Region	Asian	0.554 (0.534‐0.575)	0.886 (0.869‐0.900)	9.883 (6.672‐14.639)	0.767
		Other areas	0.744 (0.672‐0.808)	0.842 (0.769‐0.900)	15.206 (7.798‐29.653)	0.860
	Control type	HC	0.604 (0.563‐0.644)	0.938 (0.916‐0.955)	22.151 (14.827‐33.093)	0.893
		BD	0.556 (0.533‐0.579)	0.852 (0.831‐0.872)	6.990 (4.661‐10.483)	0.740
	Sample size	≥ 100	0.557 (0.533‐0.580)	0.880 (0.862‐0.897)	10.196 (6.694‐15.529)	0.770
		˂100	0.541 (0.508‐0.574)	0.911 (0.890‐0.929)	9.878 (5.667‐17.218)	0.802
	Sample source	Plasma	0.516 (0.476‐0.555)	0.934 (0.909‐0.953)	11.476 (5.024‐26.212)	0.718
		Serum	0.586 (0.563‐0.609)	0.861 (0.841‐0.880)	10.170 (6.782‐15.251)	0.800
	Assay method	MSP	0.553 (0.533‐0.574)	0.885 (0.869‐0.900)	9.483 (6.433‐13.980)	0.750
		Other methods	0.767 (0.694‐0.829)	0.848 (0.773‐0.906)	20.130 (10.035‐40.381)	0.908
	Methylation gene location	*RASSF1A*	0.644 (0.608‐0.678)	0.875 (0.847‐0.900)	12.550 (7.826‐20.126)	0.841
		Other gene location	0.535 (0.511‐0.559)	0.886 (0.867‐0.904)	10.031 (6.266‐16.058)	0.750

Abbreviations: 95% CI: 95% confidence interval; AUC: area under the curve; BD, benign live diseases; ctDNA, circulating tumor DNA; DOR, diagnostic odds ratio; HC, healthy controls; HCC, hepatocellular carcinoma; MSP, methylation‐specific polymerase chain reaction; RT‐qPCR, real‐time quantitative polymerase chain reaction; SEN, sensitivity; SPE, specificity.

**Table 4:** Meta‐regression of impacts of study features on diagnostic value of ctDNA for HCC

Analysis	Covariates	Coefficient	SE	P value	RDOR (95% CI)
Quantitative analysis	Control type	1.649	0.959	0.184	5.20 (0.25‐109.94)
	Sample size	0.541	1.129	0.665	1.72 (0.05‐62.41)
	Sample source	0.264	1.227	0.843	1.30 (0.03‐64.73)
	Assay method	0.679	1.108	0.583	1.97 (0.06‐67.02)
Qualitative analysis	Region	‐0.779	0.589	0.196	0.46 (0.14‐1.53)
	Control type	1.146	0.473	0.022	3.15 (1.20‐8.27)
	Sample size	0.401	0.385	0.307	1.49 (0.68‐3.28)
	Sample source	0.109	0.421	0.798	1.11 (0.47‐2.64)
	Assay method	−0.756	0.671	0.269	0.47 (0.12‐1.85)
	Methylation gene location	0.289	0.416	0.493	1.34 (0.57‐3.13)

Abbreviations: 95% CI: 95% confidence interval; ctDNA: circulating tumor DNA; HCC, hepatocellular carcinoma; RDOR: relatively diagnostic odds ratio; SE: standard error.

### 3.5 Publication bias

**Figure 6:**
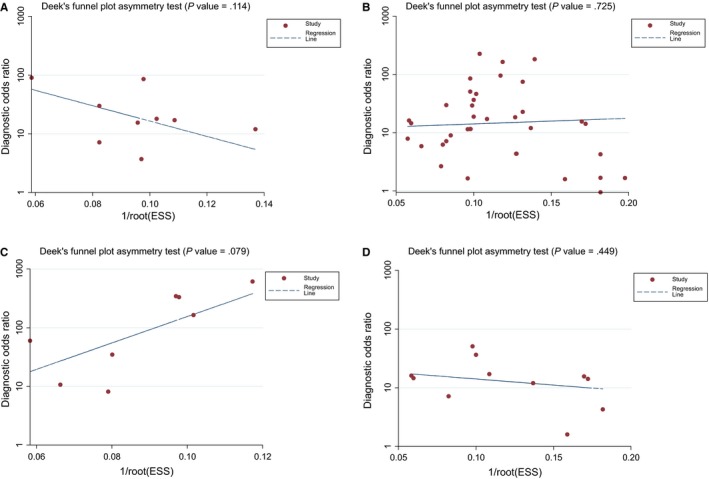
Funnel plots to evaluate the publication bias for (A) the quantitative detection subgroup; (B) the qualitative detection subgroup; (C) the subgroup of ctDNA combined with AFP assay; and (D) the RASSF1A methylation detection subgroup. AFP, alpha‐fetoprotein; ctDNA, circulating tumor DNA; DOR, diagnostic odds ratio; ESS, effective sample sizes

We examined the potential publication bias of the incorporated articles by performing Deek's funnel plot asymmetry test. Our results revealed that no significant publication bias existed in the group of quantitative analysis (Figure 6A, *P* = .114), in the group of qualitative analysis (Figure 6B, *P* = .725), in the group of ctDNA combined with AFP assay (Figure 6C, *P* = .079), or in the group of *RASSF1A* methylation detection (Figure 6D, *P* = .449).

## 4 DISCUSSION

HCC is a high‐grade malignant neoplasm with undesirable prognosis and high mortality, which is largely attributable to its low early diagnostic rate. Therefore, it is essential to disclose novel and effective biomarkers for the detection and diagnosis of early‐stage HCC. Applying novel molecular technologies to liquid biopsies has advanced our understanding of the effect of ctDNA detection on HCC diagnosis.^1^, ^12^ In this diagnostic meta‐analysis, we aimed to incorporate these published results for the first time and systematically estimate the diagnostic accuracy of ctDNA for HCC.

In our meta‐analysis, compared with the group of quantitative analysis, the group of qualitative analysis yielded a lower SEN (0.568 vs 0.722) and AUC (0.787 vs 0.880), and that was probably because some genetic loci selected for test were predominantly expressed in non‐HCC individuals.^50^, ^62^ However, the SPE (0.882) of the qualitative group was superior to that of the quantitative group (0.823). Notably, we specifically concentrated on *RASSF1A* methylation in the qualitative analysis of ctDNA *RASSF1A* is a well‐acknowledged tumor suppressor and is continually inactivated by promoter hypermethylation in HCC. It has the capability to trigger autophagy defects to facilitate oxidative stress and genome instability, thus accelerating tumorigenesis.^67^ We revealed that *RASSF1A* methylation discriminated HCC patients from control individuals with a SEN of 0.644 and a SPE of 0.875, contributing to an improvement of AUC from 0.787 to 0.841. These results indicate that circulating tumor DNA *RASSF1A* methylation can serve as a potential biomarker to screen HCC.

Our results also showed that AFP, as the most frequently used biomarker for HCC diagnosis, exhibited an unsatisfactory diagnostic performance on account of a low SEN of merely 0.478, which was relatively lower than the result of Farinati et al (the SEN was 0.540).^68^ Thus, quantitative or qualitative analysis of ctDNA was more sensitive and feasible, and the diagnostic accuracy of ctDNA was superior to the AFP assay alone (the AUC was merely 0.638). Additionally, the combined detection of ctDNA and AFP assay resulted in a remarkably increased diagnostic accuracy with a SEN of 0.760 and a SPE of 0.920 as well as an AUC of 0.944 in discriminating HCC from control individuals. This encouraging result highlights that the combined AFP and ctDNA assay for diagnosing and evaluating HCC can generate much more favorable accuracy than does either method on its own and that ctDNA detection potentially develops into a novel auxiliary tool for AFP in the screening and detection of HCC.

Furthermore, we also analyzed the DOR to evaluate the diagnostic accuracy in each group. The discriminatory test performance would be considered satisfactory when the numerical value of DOR was higher than 10.^25^ In our results, the DOR for quantitative and qualitative ctDNA assay to distinguish HCC cases from control subjects was 18.532 and 10.457, respectively. The DOR of *RASSF1A* methylation detection of ctDNA (12.550) was slightly higher than that of qualitative ctDNA assay (10.457). While the DOR for AFP assay to discriminate HCC and controls was much lower at 6.284. The DOR was dramatically improved to 54.864 when utilizing the combined detection of ctDNA and AFP, indicating a powerful capability of integrating ctDNA analysis with AFP to exactly screen and diagnose HCC.

In our report, the value of PLR in quantitative and qualitative detection of ctDNA was 4.208 and 4.378, respectively, manifesting that HCC cases have an approximately four to five fold higher chance of being ctDNA assay‐positive in comparison with control individuals. Compared with the quantitative detection of ctDNA, the qualitative detection of ctDNA displayed a higher NLR (0.489), implying that the probability for cases with negative qualitative assay results to have HCC is 48.9%. Thus, a negative ctDNA test result should be explained prudently when single‐gene methylation is independently utilized to screen and detect HCC. Nevertheless, addition of AFP statistically boosted the overall accuracy and robustness, with a PLR of 9.469 and an NLR of 0.234.

Publication bias was not revealed in our meta‐analysis by formulating Deek's funnel plot. Furthermore, a meta‐regression analysis was performed to explore the potential source of heterogeneity, thus demonstrating that in these quantitative studies, none of the parameters (such as sample source, sample size, control types and assay methods) represented a primary source of heterogeneity. Heterogeneity might have arisen because of additional reasons, including enrolled patients’ age, tumor size, lymph node invasion, lesion metastasis, TNM staging and discrepancies in the surgical protocol, which failed to be evaluated in this study on account of partial deficiency of the data or illegible details. Furthermore, the covariate of “control types” potentially exerted certain influence on heterogeneity in the qualitative analysis group. Therefore, further large clinical trials should reasonably select control individuals to boost the diagnostic performance of ctDNA in HCC.

Notably, several limitations deserve to be discussed in our meta‐analysis. Firstly, in spite of the thorough literature search, we did not incorporate several valuable articles because we failed to access their full texts. Moreover, a relatively smaller number of publications were incorporated into the quantitative group, thus potentially diminishing the statistical significance. Thirdly, some bias was potentially generated in this analysis because we merely included English‐language articles. Ultimately, we failed to include some covariates that were not depicted in these included studies, such as neoplasm size, lymph node invasion, lesion metastasis, and TNM staging of tumors. Therefore, more large‐scale prospective clinical researches that delineate the diagnostic value of ctDNA detection for HCC are needed to further identify the conclusions of this meta‐analyses.

## 5 CONCLUSION

In summary, we performed the first integrated meta‐analysis on the overall diagnostic accuracy of circulating tumor DNA assays in HCC. The diagnostic performance of quantitative and qualitative analysis of ctDNA was superior to the classical HCC biomarker AFP. Specifically, ctDNA *RASSF1A* methylation potentially serves as an effective diagnostic biomarker for HCC. Notably, because of deficiency of robustness, the ctDNA assay cannot be utilized as an independent diagnostic tool. The combined assays of ctDNA and AFP yielded a higher level of discriminatory power in HCC detection. Therefore, quantitative and qualitative analysis of ctDNA can be used as a complementary strategy integrated with AFP assay for the early detection and diagnosis of HCC. Larger sample studies are needed to further confirm our conclusions and to make the ctDNA approach more sensitive and specific.

## CONFLICTS OF INTEREST

The authors declare that there are no conflicts of interest.

## AUTHOR CONTRIBUTIONS

PG Cao, ZY Zhang designed/planned the study and wrote the paper. ZY Zhang, P Chen, H Xie performed the computational modeling, acquired and analyzed clinical data. H Xie and P Chen performed the imaging analysis. ZY Zhang, H Xie, P Chen, PG Cao participated in discussion of related data.
