# Supplementary material for: Using circulating tumor DNA as a novel biomarker to screen and diagnose hepatocellular carcinoma: A systematic review and meta‐analysis
Source: Cancer Med. 2019 Dec 26;9(4):1349–64. doi: 10.1002/cam4.2799 (PMC7013058; doi:10.1002/cam4.2799)
Supplement: Supplementary file 1 [file CAM4-9-1349-s001.pdf]

	Risk of Bias				Applicability Concerns		
	Patient Selection	Index Test	Reference Standard	Flow and Timing	Patient Selection	Index Test	Reference Standard
Chang 2008	?	?	+	+	+	+	+
Chu 2004	+	+	+	+	+	+	+
Dong 2008	+	?	+	+	+	–	+
Dong 2015	?	+	+	+	+	+	+
Dong 2017	+	+	+	+	+	+	+
Gai 2018	+	+	?	+	+	–	+
Han 2014	?	+	+	–	–	+	+
Hu 2010	+	?	+	+	–	+	+
Hu 2017	?	–	+	+	+	+	+
Huang 2011	+	+	+	+	+	+	+
Huang 2012	?	?	+	+	+	+	+
Huang 2014	?	+	+	?	+	–	+
Huang 2015	+	?	+	+	–	?	+
Iizuka 2011	?	?	+	+	+	+	+
Ji 2014	+	+	+	+	+	+	+
Kisiel 2019	–	+	+	+	+	+	+
Kuo 2014	?	+	+	–	+	+	+
Li 2014	+	?	+	+	–	+	+
Lin 2005	+	+	+	+	+	+	+
Mansour 2017	–	+	+	?	+	+	+
Mohamed 2012	–	?	+	+	+	+	+
Ren 2006	+	+	+	+	+	–	+
Sun 2010	+	?	+	+	–	+	+
Sun 2013	?	?	+	+	+	+	+
Wang 2006	–	?	+	?	–	+	+
Wei 2018	?	?	+	+	+	+	+
Wong 2000	+	–	+	+	–	+	+
Wong 2003	+	?	+	+	+	+	+
Yang 2011	–	?	+	+	+	+	+
Yang 2014	?	?	+	–	+	+	+
Yeo 2005	–	?	+	+	+	+	+
Zhang 2007	+	+	+	+	+	+	+
Zhang 2013	?	?	+	+	+	–	+

– **High** ? **Unclear** + **Low**
