# Supplementary figures and images for: Using circulating tumor DNA as a novel biomarker to screen and diagnose hepatocellular carcinoma: A systematic review and meta‐analysis
Source: Cancer Med. 2019 Dec 26;9(4):1349–64. doi: 10.1002/cam4.2799 (PMC7013058; doi:10.1002/cam4.2799)

A

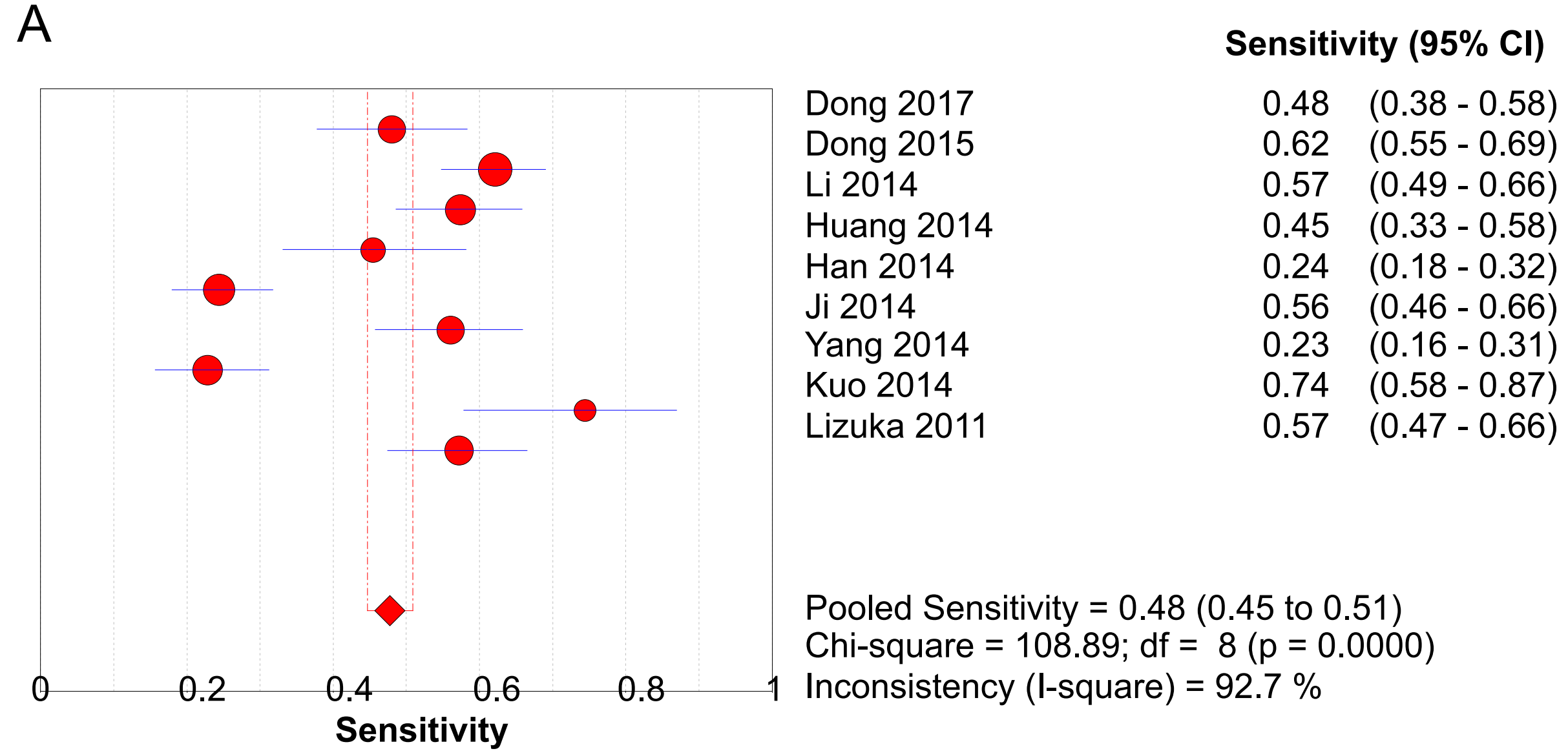

B

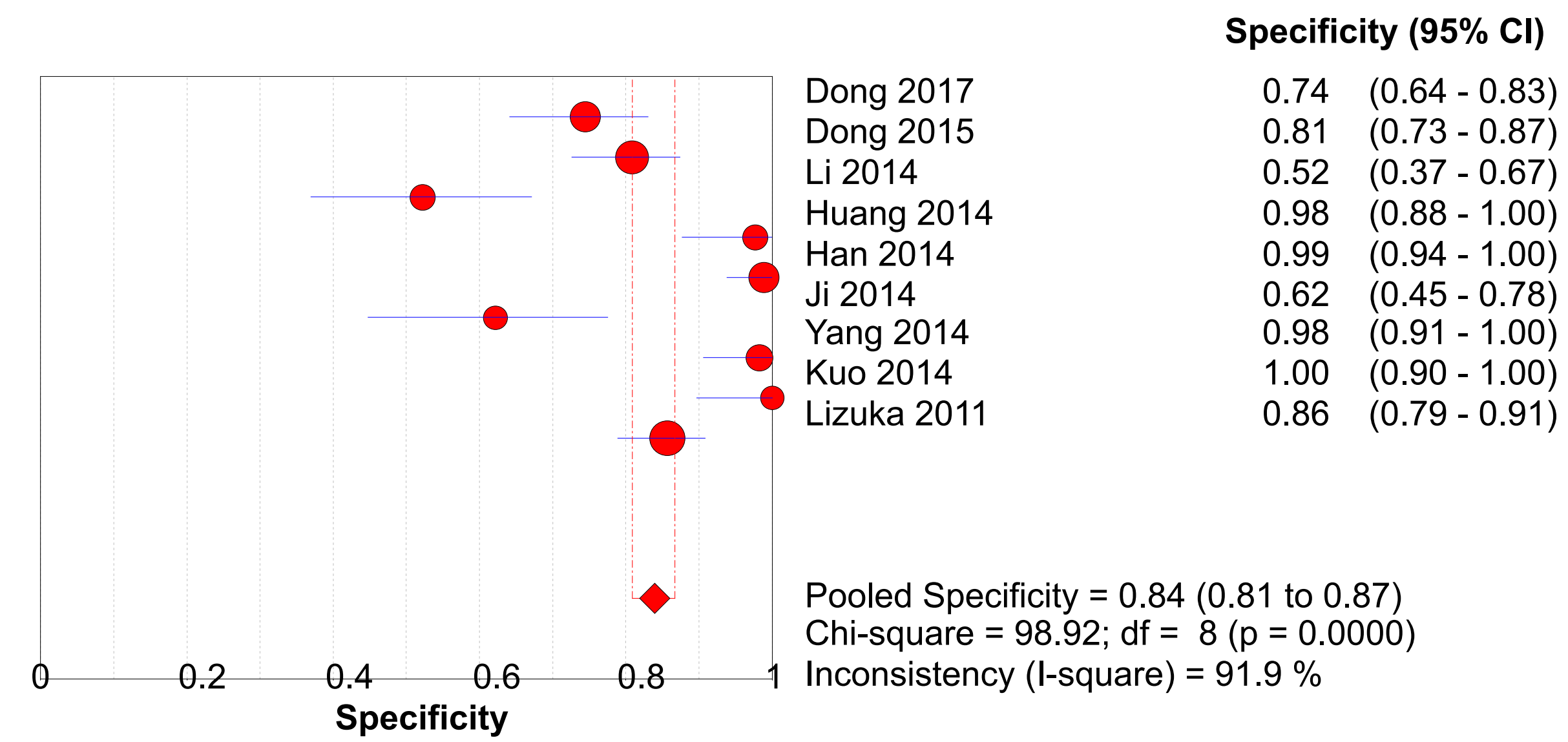

C

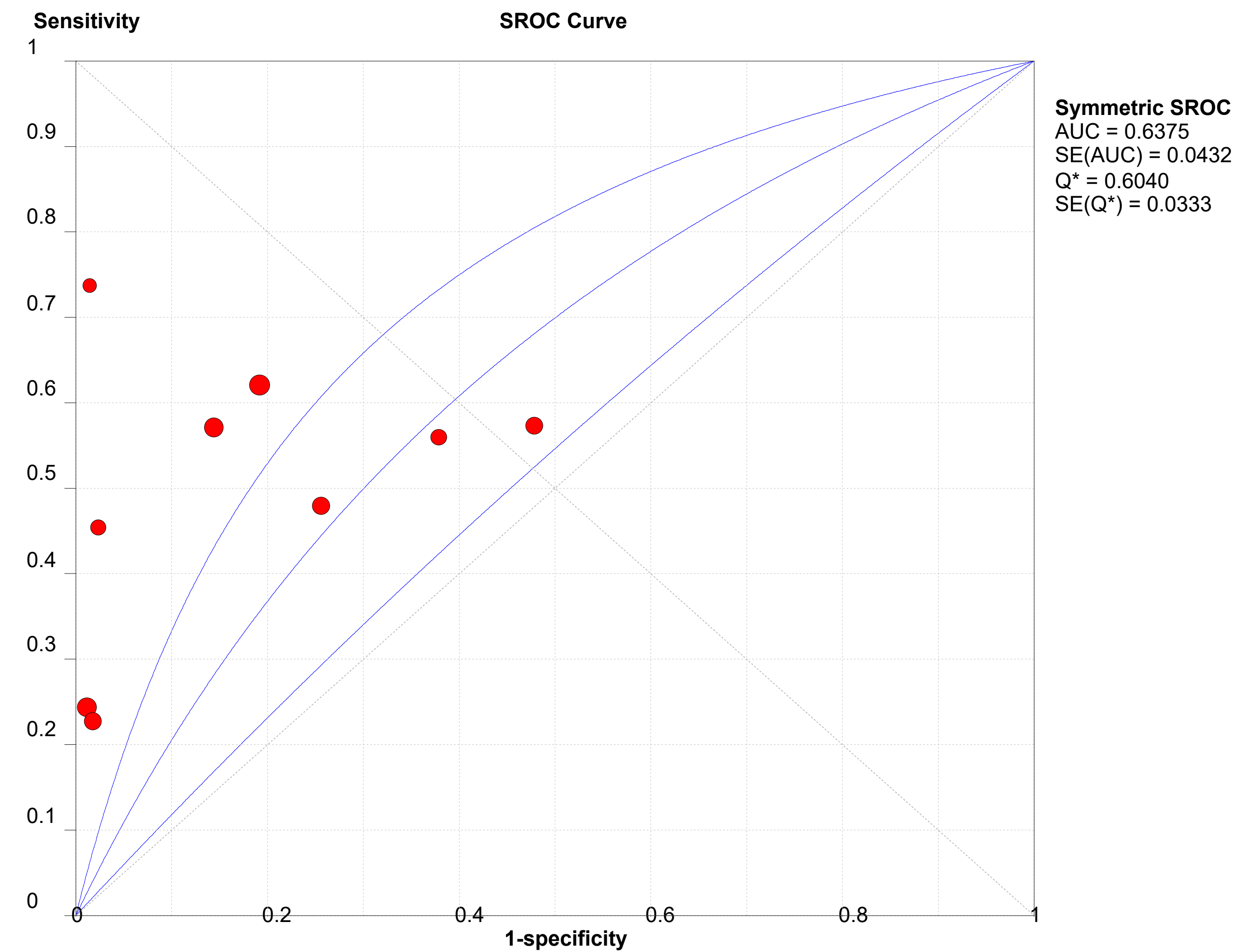

Supplement: Supplementary file 2 [file CAM4-9-1349-s002.pdf]
